# Escitalopram for Agitation in Alzheimer’s Dementia

**DOI:** 10.1002/alz.085738

**Published:** 2025-01-09

**Authors:** Tarek K Rajji, Sheriza Baksh, David Shade, Zahinoor Ismail, Amer M. Burhan, Hamid Okhravi, Prasad R Padala, Paul B. Rosenberg, Lon S. S. Schneider, Anton P. Porsteinsson, Constantine G. Lyketsos

**Affiliations:** ^1^ Centre for Addiction and Mental Health, Toronto, ON Canada; ^2^ Johns Hopkins, Baltimore, MD USA; ^3^ Johns Hopkins University School of Medicine, Baltimore, MD USA; ^4^ Hotchkiss Brain Institute, University of Calgary, Calgary, AB Canada; ^5^ Ontario Shores Centre for Mental Health Sciences, Whitby, ON Canada; ^6^ Eastern Virginia Medical School, Norfolk, VA USA; ^7^ University of Arkansas for Medical Sciences, Little Rock, AR USA; ^8^ Keck School of Medicine of USC, Los Angeles, CA USA; ^9^ University of Rochester School of Medicine and Dentistry, Rochester, NY USA; ^10^ School of Medicine, Johns Hopkins University, and Johns Hopkins Bayview Medical Center, Baltimore, MD USA

## Abstract

**Background:**

Agitation is a common and disabling symptom of Alzheimer’s dementia (AD). Pharmacological treatments are recommended if agitation is not responsive to psychosocial intervention. Citalopram was effective in treating agitation in AD but was associated with cognitive and cardiac risks linked to its R‐ but not S‐enantiomer. Primary objective of the Escitalopram for Agitation in Alzheimer’s Disease (S‐CitAD) study was to examine the efficacy and safety of escitalopram, in combination with PSI, for the treatment of agitation in patients with AD who fail to respond to psychosocial intervention. A secondary objective was to define groups with agitation most likely to benefit from escitalopram.

**Method:**

S‐CitAD is an NIH‐funded, multicenter, community‐based, phase 3, double‐masked randomized placebo‐controlled trial. Assessments were conducted at enrollment, following three weeks of psychosocial intervention (baseline), and then weeks 1‐4, 6, 7, 9, 10 and 12. Participants were adults with AD, a telephone Mini‐Mental State Examination Telephone score from 3 to 20, and clinically significant agitation/aggression as assessed by the Neuropsychiatric Inventory with either frequency being “Very frequently”, or frequency being “Frequently” and severity being “Moderate” or “Marked”. Following three weeks of psychosocial intervention alone, participants not showing response were randomized (1:1) to receive escitalopram at a target dose of 15mg/day or placebo for 12 weeks while continuing PSI. Participants who responded to three weeks of psychosocial intervention alone continued to receive the intervention and were monitored on usual care through week 12. Primary outcome was the proportion of participants with clinically significant improvement in agitation from baseline on the modified Alzheimer’s Disease Cooperative Study ‐ Clinical Global Impression of Change (mADCS‐CGIC).

**Result:**

Two hundred and five (N = 205) participants were enrolled, and 173 were randomized to escitalopram or placebo for 12 weeks while continuing psychosocial intervention. The last participant will complete the study in late winter and mask will be broken in March 2024. Outcomes will be first presented at the AAIC meeting.

**Conclusion:**

As escitalopram is a most commonly used treatment for disruptive behavior in dementia, the outcomes whether positive or negative will have major clinical impact.

